# “Airplane ear”—A neglected yet preventable problem

**DOI:** 10.3934/publichealth.2019.3.320

**Published:** 2019-08-26

**Authors:** Sudip Bhattacharya, Amarjeet Singh, Roy Rillera Marzo

**Affiliations:** 1 Department of Community Medicine, Himalayan Institute of Medical Sciences, Dehradun, India; 2 Department of Community Medicine, School of Public Health, Postgraduate Institute of Medical Education and Research, Chandigarh, India; 3 Department of Public Health, Asia Metropolitan University, Johor Bahru, Malaysia

**Keywords:** airplane ear, earache, valsalva, health promotion, prevention

## 1. Introduction

**Figure 1.**
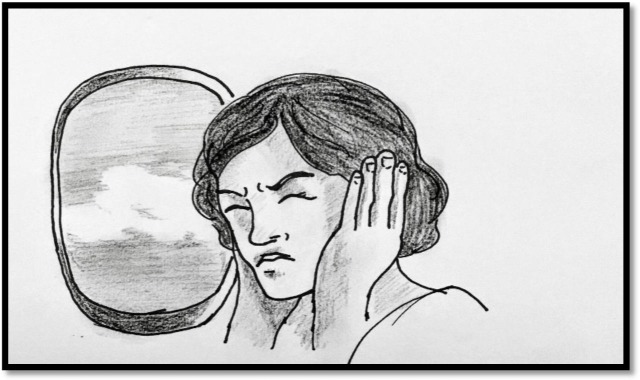
Airplane ear.

Airplane ear or ear discomfort during flight is common irrespective of ticket price we pay for our flights according to class. Whether we get extra leg space or extra facilities during flying in business class, air travelers often face this problem of airplane ear (Figure 1). Airplane ear is also known as ear barotrauma, barotitis media or aerotitis media [1]–[5]. Severe earache affects individuals of varying levels of social determinants of health as it is entirely a physiological phenomenon [3]. Airplane ear is commonly experienced when the airplane is ascending or descending, which makes it one of the commonest health problems for people travelling in air routes.

## 2. Etiology

Generally, airplane ear occurs due to quick changes in altitude and quick changes in air pressure [4]. In normal physiological conditions, the air pressure in our middle ear cavity is nearly equivalent to that of the external ear canal. This equalization of air pressure is maintained physiologically and contributes to regular auditory function and maintenance of normal balance among individuals.

However, the eustachian tube dysfunction may occur with any changes in pressure and failure to ventilate through the middle ear space. This may result in outward bulging of the tympanic membrane causing moderate to severe earache. This phenomenon can be compared with a bread expanding while baking [3],[5].

**Figure 2.**
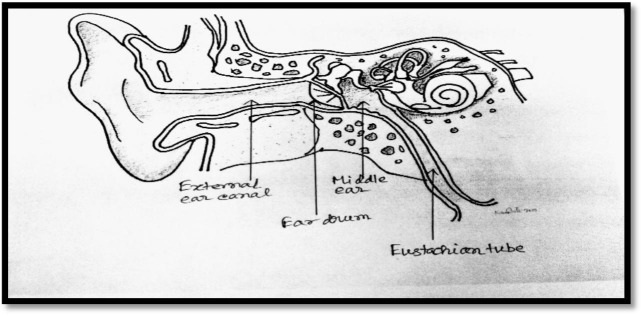
Imbalance of air pressure in the middle ear.

Conversely, due to the vacuum effect, if the air pressure inside the middle ear space reduces rapidly compared to the external ear pressure, the tympanic membrane may be pulled inside due to the pressure gradient. The eustachian tube becomes flattened during the pressure changes and it necessitates bringing air into the middle ear (Figure 2). During sudden ascend or descend of an aeroplane, ear cavity pressure is often decreased complemented by an increase in the cabin compared to the outside air pressure. In such a scenario, the unusual stretching of the eardrum or tympanic membrane may precipitate pain in the ear. At the same time, individuals may also experience decreased hearing abilities and muffled sounds as the eardrum as it becomes unable to vibrate normally [2]–[4].

The pathophysiology remains the same in cases of scuba diving, in hyperbaric oxygen chambers, and during explosions happening nearby [2].

## 3. Symptoms [3]–[5]

Airplane ear can in occur unilaterally or in both ears. Signs and symptoms of Airplane air include discomfort, pain, and fullness in ear, and mild to moderate hearing loss in acute cases. Moreover, for severe cases, affected individuals may experience severe pain, moderate to severe hearing loss, tinnitus, vertigo, and hemotympanum (severe form).

## 4. Risk factors [3]–[5]

The common risk factors for airplane ear include—small eustachian tube (infants and toddlers), common cold, acute or chronic sinusitis, allergic rhinitis, otitis media, and napping on an airplane during rapid pressure change in our middle ear. Permanent damage may occur in the membranous linings of the middle ear or eustachian tube, which aggravates the problem further [6].

## 5. Complications

Generally, airplane ear does not pose problems unless there is any structural damage in the middle ear. Rare complications may include permanent hearing loss and chronic tinnitus [6],[7].

It is very surprising that despite being a very common condition, no prior preventive instruction is given by the air hostesses/airplane authority about this phenomenon. Although the airline authorities provide a lot of instructions to the passengers regarding emergency landing in the water, power failures, low oxygen supply to the cabin and so on; airplane ear remains a neglected health issue in aerospace safety measures.

Furthermore, if a baby suddenly cries or elderly adults feel ear discomfort or pain during rapid descent of ascent of an airplane, then the flight attendants would rush to the sufferers and offer instructions/help. Sometimes, they may have to manage emergencies like ear bleeding by apply ear packs. All these health hazards and associated challenges can be easily prevented if airplane authorities include following preventive instructions for airplane air with existing life-saving instructions.

## 6. Prevention [2],[3],[5]–[15]

### 6.1. Primary prevention

(a). Yawning, chewing and swallowing is helpful: During take-off and landing chewing gum and swallowing activate the muscles that make patent the eustachian tubes bi-laterally.
(b). Don't sleep during ascent and descent of flights: If we are awake during the period of sudden pressure change, we can practice health promotive measures whenever we feel discomfort on our ears.
(c). Reschedule travel plans: If possible, it is better to not travel by plane if an individual is suffering from the common cold, sinusitis, nasal congestion, recent ear surgery or ear infection.
(d). Use of earplugs: Earplugs slowly equalize the pressure against our eardrum during take-off and landing of airplane.
(e). Use of decongestants: It is helpful if taken before 30 minutes to an hour before the travel.

### 6.2. Secondary prevention[7]–[15]

**Figure 3.**
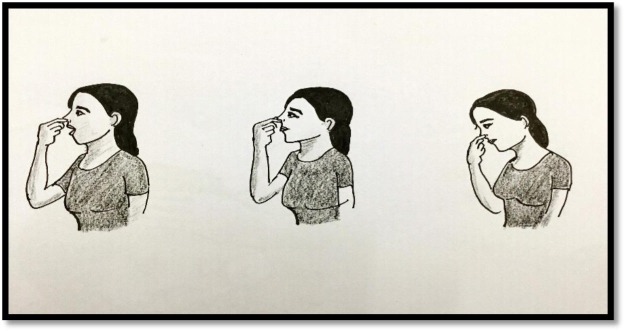
Valsalva manoeuvre.

(a). Valsalva manoeuvre: The valsalva manoeuvre can be performed by the passengers during passengers feel ear discomfort in the aeroplane. It (Figure 3) is performed by moderately forceful exhalation against the closed glottis. Commonly it is done by closing one's mouth, pinching one's nose shut while pressing out as if blowing up a balloon. This manoeuvre is often used to clear the ears and sinuses (that is to equalize pressure between them) during ambient pressure changes. It helps to maintain the air pressure in the middle air, by contracting several muscles in the pharynx to elevate the soft palate and open the throat. Especially, the muscle, tensor veli palatini, also acts to open the eustachian tube and sucking or bulging of eardrum is prevented/corrected during pressure changes in the flight.
(b). Bleeding ear: In case of bleeding, immediate ear packing is often recommended, followed by early exploration in the OT under expert physician is required.

### 6.3. Tertiary prevention

For frequent fliers, surgically placed tube in the eardrums is generally helpful to aid fluid drainage in the middle air, particularly for those who are prone to severe airplane ear. This tube helps in ventilating air to the middle ear, and equalize the pressure between the outer and middle ear.

## 7. Conclusion and recommendations

Airplane ear is a common yet, ignored public health problem. It can be handled effectively if proper precautions/corrective measures are adopted. Previously in the airlines, lozenges were offered, which may have helped the passengers to avoid this problem, but now a days it is less commonly practiced. The airplane authority should explore socio-culturally appropriate and evidence-based strategies and incorporate them with the preventive and curative instructions for the passengers. Moreover, the flight attendants may be trained in recognizing and managing airplane ear alongside other life-saving instructions to their passengers. It may decrease the air discomforts and empower the air passengers during air travel.
